# Advances in Anther Culture-Based Rice Breeding in China

**DOI:** 10.3390/plants14111586

**Published:** 2025-05-23

**Authors:** Xinxing Chen, Sanhe Li, Wenjun Zha, Changyan Li, Lei Zhou, Aiqing You, Yan Wu

**Affiliations:** 1 Hubei Key Laboratory of Food Crop Germplasm and Genetic Improvement, Key Laboratory of Crop Molecular Breeding, Ministry of Agriculture and Rural Affairs, Institute of Food Crops, Hubei Academy of Agricultural Sciences, Wuhan 430064, China; 2023301120199@webmail.hzau.edu.cn (X.C.); lisanhe1@hbaas.com (S.L.); zwj_19850202@hbaas.com (W.Z.); lichangyan@hbaas.com (C.L.); zhoulei@hbaas.com (L.Z.); 2 College of Plant Science and Technology, Huazhong Agricultural University, Wuhan 430070, China; 3 Hubei Hongshan Laboratory, Wuhan 430070, China

**Keywords:** rice (*Oryza sativa* L.) anther culture breeding, influencing factors, current status of research, prospect

## Abstract

The anther culture-based breeding of rice is a plant tissue culture technique that utilizes rice pollen to rapidly obtain haploid plants. In comparison with traditional breeding methods, this technique shortens the breeding cycle and enables the quick generation of homozygous plants, which is of great significance for the development of new rice varieties and the expansion of germplasm resources. With the advancement of technologies, the use of the anther culture technique in rice breeding has matured and has been applied to the development and utilization of new varieties with high yield, multiple resistances, and superior quality, in combination with other breeding methods. This technique has gained widespread attention globally, with many countries adopting it to create new germplasm resources. This study reviews advances in the rice anther culture technique, the factors influencing anther culture efficiency, and the progress in breeding rice varieties using this technique, as well as analyzes the current challenges and future prospects of anther culture breeding.

## 1. Introduction

Rice (*Oryza sativa* L.) plays a pivotal role in global food production [1] and stands as one of China’s four major grain crops, critically contributing to national food security. By 2050, the global population is projected to reach 10.6 billion, leading to an increased demand for rice [2]. In view of decreasing arable land and rising grain demands, it is essential to continuously enhance rice yield per unit area while maintaining or improving the rice quality. It is also crucial to surpass the limitations of traditional rice breeding, employ genetic improvement methods to develop novel breeding techniques, and create new varieties that can bear biotic stresses—such as multiple diseases and pests [3]—and abiotic stresses—like drought and flooding [4]. These efforts are vital to meet people’s ever-growing needs for a better life, ensure food security, and support sustainable agricultural development in China.

In rice production, traditional breeding methods require five to six generations of self-crossing or backcrossing to obtain sufficiently homogenized inbred lines—a process that is labor-intensive, time-consuming, and costly. To address critical challenges in crop improvement—including accelerated breeding cycles, expanded genetic variation, and enhanced selection efficiency—researchers have developed anther culture-based rice breeding as an innovative methodology within modern agricultural biotechnology. Rice anther culture involves the in vitro culture of rice anthers or pollen grains to produce callus tissue formed by microspores and then differentiate into whole plants. With ongoing research and refinement of anther culture techniques, a combination of traditional breeding, gene editing, and molecular marker-assisted selection with anther culture can significantly enhance rice yield and quality [5,6], shorten growth duration, and improve stress resistance [7], which is vital for the production and development of new rice varieties.

This paper aims to study the current rice anther culture technique and its applications, including the progress and influencing factors, as well as advances, challenges, prospects and summaries of anther culture breeding, which provides a theoretical reference for the enhancement and application of this technique.

## 2. Advances in Rice Anther Culture Technique

In 1968, Japanese researchers successfully obtained the first haploid rice plant using the anther culture technique [8], marking a new era for rice breeding [9]. In China, rice anther culture began in 1970. The successful creation of the first new japonica rice variety “Danfeng 1” via this technique in 1975 signified the practical application of anther culture in rice breeding [10]. In 1974, the N6 medium for anther culture was originally developed in China [11], laying the foundation for subsequent research on rice anther culture. Since then, over 40 new varieties have been bred using anther culture, e.g., Jinghua, Zhonghua, and Longgeng series [12]. However, few indica rice varieties were developed utilizing this technique and, by the end of the 20th century, just five varieties were bred in Jiangxi with small promotion areas [8]. The anther culture technique has matured and become an important biotechnological tool for the rapid propagation and preservation of rice germplasm resources [13].

Using the anther culture technique in rice breeding provides a rapid pathway to achieve plant homozygosity and homogeneity [14,15,16,17]. Currently, rice anther culture involves two major steps—callus induction and plantlet differentiation. The efficiency of anther culture varies among different rice varieties and under different culture conditions, typically measured by callus induction and green plantlet differentiation rates [18]. For example, Yu Bo et al. [19] used anther culture to complement favorable traits between Chinese and American rice parents and obtained 18 improved Chinese rice germplasms and 6 improved American rice germplasms. Weifeng et al. [20] developed a new rice variety, “Chongshang 2022”, with lodging resistance, blast resistance, wide adaptability, and superior quality via a combination of anther culture and molecular marker-assisted selection. In the future, more new germplasms will be created based on anther culture technique, driving the continuous advancement of rice variety development in China.

## 3. Factors Affecting Anther Culture in Rice

**Figure 1:**
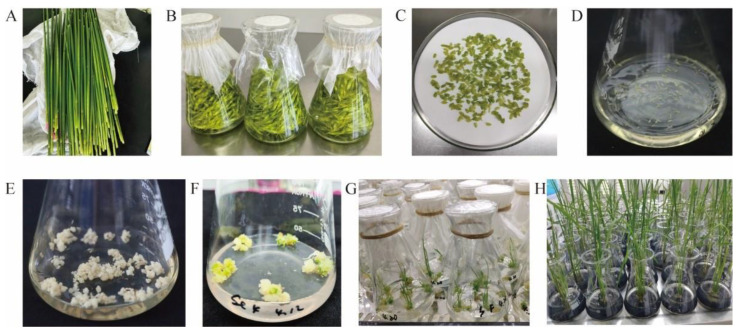
Process of anther culture in rice. (**A**): Sampling of rice spikes and low-temperature pretreatment; (**B**): Disinfection of spikelets; (**C**): Taking anthers for inoculation; (**D**): Inoculation of anthers onto induction medium; (**E**): Callus inducted from anthers; (**F**): Differentiation of callus tissue; (**G**): Plantlet regeneration from callus; (**H**): Seedlings derived from anther culture.

Rice anther culture is influenced by factors such as genotype, pollen development, medium composition, culture conditions, and low-temperature treatment [21,22], making the process challenging (Figure 1). Indica rice materials, in particular, are constrained by genotype, with low anther culture efficiency and small progeny populations, which hinders breeding selection [23] and subsequent research. Thus, higher anther culture efficiency and larger progeny populations are crucial for the success of anther culture in rice breeding programs [24]. All factors in the anther culture regeneration system must be strictly controlled to maximize the efficiency of anther culture in rice.

### 3.1. Genotype

Genotype is the most important factor affecting the efficiency of anther culture in rice [25]. The efficiency of rice anther culture, modulated by genotype, is a heritable trait [26]. Genotype has a significant influence on green plantlet differentiation rate, callus induction rate, and anther culture efficiency [27,28,29]. There are significant differences in the callus induction rate and differentiation rate of rice materials with different genotypes [30]. Some studies have reported that the anther culture efficiency of different types of rice is in the following order: indica rice < indica hybrid rice < japonica–indica hybrid rice < japonica rice < glutinous rice, with significant variation even among different varieties of the same type [31]. Zou Liping et al. [32] found that the green plantlet differentiation rate was principally influenced by the additive effects of genes, while the callus induction rate was regulated by both the additive and non-additive effects of genes, both of which were quantitative traits controlled by nuclear genes and could be independently inherited [33]. The narrow-sense heritability of both callus induction rate and green plantlet differentiation rate is relatively high, and appropriate combinations of the two can effectively enhance anther culture efficiency and obtain better results.

Among the current rice types, the induction of indica rice is more challenging than that of japonica rice [34], while the efficiency of flower culture is lower than that of japonica rice [35,36]. The callus induction rate of japonica rice is generally above 10%, with the highest rate over 40% [37], and the application of the anther culture technique in japonica rice breeding programs is well established [38]. In contrast, indica rice materials generally have low anther culture efficiency, their callus induction rates are typically below 5%, with a mean rate of 0.5%, and their green plantlet differentiation rates range from 0.1% to 1.0%, with a mean rate of around 0.5% [8]. Some materials hardly produce callus and regenerated plantlets [39], and the mean plantlet regeneration rate is about 1–3% [40], resulting in small progeny populations that severely constrain the use of the anther culture technique for breeding new indica rice varieties. Previous studies have shown that anther culture efficiency varies significantly among different rice materials, with anther culture efficiency of F1 generally falling between the parents, closer to the female parent. While the anther culture efficiency of parents is high, that of F1 is also high [41,42]. Therefore, selecting japonica rice materials with high culture efficiency as the female parent can improve anther culture efficiency of F1 and the application of this technique in indica rice.

### 3.2. Pollen Development, Sampling Period, and Selection of Spikes

The developmental stage of rice pollen is one of the crucial factors influencing the efficiency of anther culture. The use of in vitro culture of pollen to obtain callus is only applicable to a specific mature stage of pollen; callus induction cannot be achieved at other developmental stages of the pollen. The process of rice pollen development can be divided into eight stages according to the research of Feng Jiuhuan et al. [43], i.e., the formation stage and meiotic stage of microspore mother cells, the early, middle, and late stages of microspores, the early and late stages of bicellular pollen, and the mature stage of pollen. Among them, the best period for anther culture in rice is the mid–late uninucleate stage, i.e., the middle and late stages of microspores when the ability to induce callus formation from pollen cells is strongest [44,45,46]. Sampling rice spikes too early or too late will affect the callus induction rate [18].

To accurately find the pollen cells at the mid–late uninucleate stage during sampling in the field [47], the morphological indicators of some organs are used to judge the developmental stage of the anther. During sampling, the main spike of rice should be selected as much as possible. When the young spike bracts of rice are large and unbroken, most glumes are near maturity, the color is yellow-green, the upmost pulvinus interval between the flag leaf and the top second leaf is 8–13 cm, and the length of the filament is 1/3–1/2 of the length of the glume, the materials can be taken [48]. Among them, the upmost pulvinus interval is the main factor affecting the callus rate of the anther [49]; however, for different types of rice materials, the morphological indicators such as the upmost pulvinus interval, the color of the glumes, and the length of the anthers may be different. Thus, in the experimental process, to ensure the accuracy of the sampling period, some materials should be taken for microscopic examination first, and then the examination results should be combined with the morphological indicators to select the materials. When most of the pollen is in the mid–late uninucleate stage, samples can be taken [50] (Figure 1A). Additionally, Rownak et al. [51] found that the callus ability of the anther was significantly different in different parts of the spikelet, and the callus ability of the anther at the base of the spike was the highest.

### 3.3. Low-Temperature Pretreatment and Anther Inoculation

In 1983, Qu Rongda et al. [52] proposed that the callus induction rate and green plantlet differentiation rate of pollen could be significantly enhanced by low-temperature pretreatment. The reason was that low-temperature treatment could maintain the physiological environment of pollen development, slow down the degeneration of pollen, initiate the development of the male nucleus, reduce the level of ethylene and increase the level of endogenous auxin [9]. After sampling in the field, the leaves of the young spikes with bracts were cut off and the leaf sheaths were retained. The surface of the young spikes was disinfected with 75% alcohol, then the disinfected young spikes were wrapped with clean, wet gauze and sealed with fresh-keeping bags. Relevant labels were attached, indicating the name of the materials, the number of samples, and the sampling time. Finally, the wrapped young spikes were stored in the refrigerator. The research showed that placing the anthers at 8–10 °C for 7–10 d achieved better anther culture results [53], and the longest time should not exceed 20 d [54] (Figure 1A). Furthermore, the optimal time and temperature for low temperature treatment of different rice materials were different [39].

For the inoculation of the anthers after low-temperature treatment, the bracts of the rice spikes were removed first on an ultra-clean workbench. Appropriate spikelets were then cut off and placed into a prepared sterile conical flask. Next, 75% alcohol was added to the flask to soak the spikelets for 1 min, and then the alcohol was poured out. The freshly prepared 3% sodium hypochlorite solution was added to completely immerse the spikelets for 23 min; during this period, the spikelets in the flask were stirred and shaken three times or more (Figure 1B). Subsequently, the sodium hypochlorite solution was poured out, the spikelets were washed with sterile water 4–5 times, and the water on the spikelets was dried with sterile filter paper in batches before starting the inoculation work [18,55,56] (Figure 1C). The glume-cutting and anther-shaking method was used to inoculate the anthers onto the callus induction medium on the ultra-clean workbench, with around 100–200 anthers per bottle of medium (Figure 1D). The type of medium, inoculation time, and inoculation material were all written on the bottle, and each treatment was repeated at least three times [24,57]. The callus induction medium after inoculation was placed in a dark incubator for culture, with a temperature of 26–28 °C. The callus was produced within 30–60 d after inoculation. The growth status of callus tissue was observed periodically, and the callus induction rate was calculated (Figure 1E). After a period of time, the light-yellow, compact callus with the size of about 2–3 mm was transferred to the differentiation medium to start the differentiation culture (Figure 1F), with a temperature of 26–28 °C, light time of 10–12 h/d, and light intensity of 1000–1500 Lx. The number of green plantlets and the green plantlet differentiation rate were calculated within 45 d of differentiation culture [55,57] (Figure 1G). The anther culture efficiency of the material can be obtained by calculating the callus induction rate and green plantlet differentiation rate of the material. The calculation formulas are as follows:

Anther culture capacity (green seedling yield) (%) = (callus induction rate × green plantlet differentiation rate) × 100

Callus induction rate (%) = (number of anthers forming callus/total number of in-oculated anthers) × 100

Green plantlet differentiation rate (%) = (number of callus differentiating into green plantlets/total number of inoculated callus) × 100

### 3.4. Selection of Medium and Hormone Ratio

Rice anther culture comprises two stages—callus induction and green plantlet differentiation. However, the nutrients required in these two stages are different, so the media are distinct, and the culture effects obtained are also different. The composition and ratio of the media have an important impact on the efficiency of anther culture in rice. During the process of rice anther culture, the utilization of many types of basic medium is widespread, like He5, SK3, N6, M8, improved M8, potato medium, and universal medium. Even though the experimental materials are the same, if the media used are different, the anther culture effects obtained will be disparate. In the callus induction stage, the most frequently used basic media are M8 and N6. Studies have found that, in the callus induction stage, He5 is suitable for indica rice, SK3 for indica–japonica hybrid rice [58,59], N6 medium for japonica rice [11], and M8 and universal medium for both indica and japonica rice [60,61]. In the green plantlet differentiation stage, M8 and MS media are usually used to culture and differentiate the callus [62]. At present, there is no all-purpose medium of rice anther culture for different rice varieties to obtain better culture results. Therefore, in practical applications, the composition and ratio of the media should be adjusted appropriately according to different varieties of rice materials to obtain the optimal anther culture effect.

In addition to the basic medium, the composition of the medium also comprises hormones, carbon sources, and organic additives. Among them, the induction and regeneration of callus are modulated by specific plant growth regulators [63], especially the balance regulation between cytokinin and auxin [64]. Thus, the category and ratio of exogenous hormones in the media have a vital impact on rice anther culture, and the reasonable dosage and ratio of plant growth regulators, such as 2,4-dichlorophenoxyacetic acid (2,4-D), 6-Furfurylamino-purine (KT), N-6-Benzyladenine (6-BA) and 1-Naphthaleneacetic acid (NAA), are of great significance to the enhancement of anther culture efficiency in rice. Generally, auxins such as NAA and 2,4-D are used as the major hormones to influence callus induction, with the latter possessing the most significant effect on callus induction; cytokinins are the dominant hormones in the callus differentiation stage, generally using KT, 6-BA, etc. Studies have shown that the number and development of callus treated with compound hormones are better than those treated with single hormones [65], and the suitable hormone categories and ratios for different materials vary greatly. Rahman et al. [66] reported that the callus induction rate was significantly improved after mixing 3.0 mg/L NAA with 1.0 mg/L 2,4-D, and the combination of 1.0 mg/L 6-BA and 1.0 mg/L 2,4-D enhanced the development effect of callus. The treatment of 10 μM Indole-3-Acetic Acid (IAA) combined with 10 μM 6-BA could facilitate callus induction, as proven in the research of Lakshmaiah et al. [67]. Guo Shuqiao et al. [68] found that the optimum hormone induction ratio was 2.0 mg/L 2,4-D, 0.2 mg/L KT, and 1.0 mg/L NAA, and the hormone ratio with an outstanding differentiation effect was 0.5 mg/L KT and 2.0 mg/L 6-BA in research involving the japonica rice variety “Wuyunjing 8” and three indica–japonica F1 hybrids as experimental materials. Xiang Fayun et al. [69] found that the callus induction effect of 2.0 mg/L 2,4-D, 1.0 mg/L KT, and 3.0 mg/L NAA was better, and the hormone ratio with excellent differentiation effect was 2.0 mg/L KT, 0.5 mg/L NAA and 0.5 mg/L 6-BA in the experiment with anthers of five indica–indica F1 hybrids as materials. Moreover, some other auxins like picloram, phenylacetic acid (PAA), NAA, and IAA can improve the callus induction rate when used alone or mixed with 2,4-D [70]. The category and concentration of auxin can also affect the development pathway of microspores. For example, IAA and NAA can directly cause anthers to form embryoids, without producing callus, whereas 2,4-D is beneficial to the formation of anther callus. Some studies have shown that a higher content of 2,4-D in the callus stage is not conducive to the differentiation of green plantlets. Guha-Mukherjee et al. [71] suggested that a combination of 0.5 mg/L 2,4-D with 2.5 mg/L NAA and 0.5 mg/L KT guaranteed a lower level of 2,4-D and a medium level of NAA, as well as high efficiency of anther culture in the two stages. The amount of 2,4-D needs to be carefully considered to ensure the efficiency of anther culture in the callus induction stage and the green plantlet differentiation stage. The above studies show that hormones play a vital role in the callus induction and green plantlet differentiation of anther culture. Therefore, in practical applications, the varieties and stages should be comprehensively considered to determine the optimal hormone ratio for obtaining higher anther culture efficiency.

In previous studies, the standard carbon source for the rice anther culture process was sucrose, with a concentration of 3–6% [54]. Subsequent studies confirmed that the type of carbon source had a significant impact on the efficiency of anther culture [72,73]. In rice, especially indica rice, the effect of sucrose as a carbon source to induce callus was inferior to maltose, and the differentiation of callus induced by maltose was also better [18,74]. The anther culture effect of the mixed use of maltose and sucrose (3% maltose + 3% sucrose) was better than that of a single carbon source, as demonstrated in the research by Zhu Yongsheng et al. [75]. The reason might be that the mixed carbon source satisfied different sugar metabolism requirements during distinct developmental stages in the process of callus induction [39]. In addition to carbon sources, the dosage and ratio of nitrogen sources also have an optimal range for different types of rice. The ratio of nitrate nitrogen and ammonium ion to ammoniacal nitrogen in the media will influence the efficiency of anther culture in rice. Generally, the ammonium ion content in the medium of japonica rice is 7 mmol/L, that of indica rice is 3.5 mmol/L, and that of indica–japonica hybrid rice is 3.5–7.0 mmol/L [54]. Moreover, some studies have shown that adding other components to the media will affect callus induction and green plantlet differentiation. For example, adding natural active substances, such as corn juice, potato extract, loofah bleeding sap, and coconut juice, and organic substances like yeast juice, sorbitol, proline, and hydrolyzed casein can significantly enhance the green plantlet differentiation rate of indica rice [76]. Adding activated carbon could significantly increase the fresh weight, plant height, root number and root length of rice, which was beneficial to the plantlet growth and differentiation in anther culture, as proven in the research by Ding Yuanfeng et al. [77]. Some researchers believe that the improvement in the callus induction rate of indica rice may result from the addition of an appropriate concentration of proline [78]. Wan et al. [79] found that the proliferation and rooting of “MR219” callus could be influenced by calcium lignosulfonate in the study of the recalcitrant indica rice variety “MR219”. Rakesh et al. [80] proved that polyamines could induce pluripotency, achieve molecular signal transduction, facilitate differentiation, and increase cell division in the physiological process of plants. Several studies have shown that polyamines effectively improve the plantlet formation rate and callus regeneration ability of wolfberry [81].

### 3.5. Plantlet Strengthening and Hardening and Contamination Protection

Strengthening plantlets is an important link in rice anther culture. When the green plantlets had grown to about 2–3 cm, they were moved to the rooting medium for around 7 d to strengthen the seedlings. The bottles were uncapped, and the hardening of the seedlings was performed for 3 d when the height of the seedlings was around 15 cm and the root length was about 1.5–2.5 cm. After hardening, the medium on the root was removed, and the roots and leaves were trimmed. New roots and leaves grew after raising the seedlings with clean water for 3–5 d, after which they were transplanted into the soil for conventional management (Figure 1H). Throughout the process, a suitable environmental temperature and humidity should be guaranteed, generally with the temperature at about 25 °C and the humidity at about 85%. Additionally, contamination should be prevented in the process of anther culture. For example, after sampling in the field, the materials should be disinfected before low-temperature storage. During the process of anther inoculation and subsequent transfer of callus and green seedlings, the inoculation tools and environment should be kept sterile and free of toxins to prevent contamination.

## 4. Application of Anther Culture in Rice Breeding

Rice anther culture breeding is a practical technique that utilizes plant tissue culture methods integrated with systematic selection and hybrid breeding approaches to develop new varieties. Through this technology, breeders have successfully developed photosensitive sterile lines and restorer lines for indica rice, japonica rice, and hybrid rice varieties. These achievements essentially encompass all existing types of cultivated rice species (Table 1). At present, more than 100 new rice varieties have been developed through anther culture in China. In addition, the use of the anther culture technique is widespread internationally, with reports of new lines created using this technique from India, Japan, South Korea, Hungary, and America [82]. These new varieties are not only stably obtained through anther culture but also possess excellent rice quality characteristics, such as resistance to rice blast, brown planthopper, and abiotic stress. This helps achieve the important goals of high yield, superior quality, and resistance to abiotic and biotic stresses in rice breeding programs [83].

The first conventional rice variety developed in China using the anther culture technique was the japonica rice “Danfeng 1”. Subsequently, an increasing number of rice varieties were bred via this technique, e.g., “Hejiang 21”, “Longgeng 1, 3, 4”, “Zhonghua 8, 10”, etc. Further, these new germplasms were utilized as parents to breed the Longhua, Longgeng, and Longxuan series [84]. In Liaoning Province, saline–alkali-tolerant varieties such as “Yangeng 22”, “Huageng 15”, and “Huageng 45” were developed through anther culture, creating numerous new germplasms. However, due to the poor anther culture efficiency of indica rice, its overall anther culture breeding was far inferior to that of japonica rice, with fewer varieties developed and limited cultivation areas [85]. With advancements in the anther culture technique, combined with molecular marker-assisted selection and gene editing, it has been effectively utilized to breed improved rice varieties exhibiting enhanced resistance to biotic stresses (such as pests and diseases) and abiotic stressors (particularly saline–alkaline soil conditions) [20,86,87]. Countries worldwide have fully utilized anther culture technique to create new germplasm resources. For instance, India developed “Satyakrishna” (CR Dhan 10) [88] and “Phalguni” (CR Dhan 801) [89] through anther culture, both of which exhibited lodging resistance and high yield, suitable for irrigated and rainfed shallow lowland ecosystems and irrigated wetland ecosystems, respectively. Additionally, the salt-tolerant rice variety IR51500AC11-1, obtained through anther culture, was commercialized as PSBRc50 “Bicol” for cultivation in saline-damaged paddy fields [90]. These studies demonstrate the widespread applications of anther culture in improving rice quality, enhancing yield, developing resistance, and creating new germplasm resources. Moreover, anther culture has facilitated the construction of permanent mapping populations for molecular markers in rice [91].

The anther culture technique also plays a significant role in hybrid rice breeding by accelerating the purification and selection of restorer lines, sterile lines, and maintainer lines [92], thereby enabling the development and utilization of various breeding resources. Since the 1990s, anthers of photoperiod–thermo-sensitive genic male sterile (PTGMS) lines in the two-line hybrid rice system and indica–japonica F1 hybrids have been the two primary materials for anther culture breeding. In the early 1980s, the Yangzhou Academy of Agricultural Sciences used the anther culture technique to purify and rejuvenate the indica-type three-line sterile line “Zhenshan 97A”; the indica-type sterile line “Xieqingzao A” was successfully purified in 1988; the Indonesian Shuitiangu sterile line II-32A was purified in 1990 [93]. By applying anther culture to six indica–japonica single-cross (multiple-cross) hybrids, Zhang Anzhong et al. [94] found that stable wide-compatibility restorer lines and new wide-compatibility lines could be obtained within a short period (3–4 years), indicating that rapid screening of restorer lines could be achieved through anther culture of indica–japonica F1 hybrids. Zhou Yuanchang et al. [95] demonstrated that the genetic purification of Peiai 64S was influenced by anther culture, with the resulting progenies exhibiting stable fertility. The japonica PTGMS line 1647S was developed by Liu Jianping et al. [96] using the anther culture technique, showing stable sterility under 15 h long-day conditions and excellent comprehensive traits. Li Xin et al. [97] studied seven indica PTGMS lines and F1 hybrids and found that the callus induction rate and green plantlet yield of PTGMS rice were higher than those of indica–japonica F1 hybrids, indica–indica hybrids, and conventional indica varieties. Additionally, the progenies of conventional varieties obtained from anther culture exhibited similar frequencies of haploid, diploid, and polyploid plants with PTGMS lines.

**Table 1:** Rice varieties obtained from anther culture.

Type	Variety Name	Traits	Country	References
Conventional japonica rice	Danfeng 1	Superior quality, high yield	China	[98]
	Zhonghua 8, Zhonghua 9	Rice blast resistance	China	[99]
	Zhonghua 10	Superior quality, saline-alkaline tolerance	China	[100]
	1647S	Excellent overall performance	China	[96]
	Huageng 45	Saline-alkaline tolerance, lodging resistance, bacterial blight resistance, moderate resistance to rice anthracnose, sheath blight, and false smut	China	[101]
	Hejiang 21, Longgeng 1, Longgeng 3, Longgeng 4, Longgeng 7, Longgeng 8	Rice blast resistance, superior quality, high yield	China	[84]
	Jiudao 26	Moderately resistant to leaf blast, moderately susceptible to panicle blast, superior quality	China	[102]
	Zhonghua 15	Resistance to bacterial blight and rice blast, high yield	China	[103]
	Huageng 15	Saline-alkaline tolerance	China	[104]
	Zhonghua 14, Zhonghua 16	Saline-alkaline tolerance, drought resistance, lodging resistance	China	[105,106]
	Longgeng 10, Longgeng 12	Rice blast resistance, superior quality	China	[107,108]
	Huayu 13	Resistance to rice blast, sheath blight and false smut, saline-alkaline tolerance, superior quality, good taste, high yield	China	[109]
	HD27	Superior quality, disease resistance, early flowering	China	[110]
	Chongshang 2022	Rice blast resistance, lodging resistance, good quality	China	[20]
Conventional indica rice	Shuhui 162	Rice blast resistance, superior quality	China	[111]
	Hua 1B	Good outcrossing characteristics, high combining ability	China	[112]
	Bicol (IR51500AC11–1)	Saline-alkaline tolerance	Philippines	[90]
	CR Dhan 10 (CRAC2221–43), Satyakrishna	Resistance to neck blast, sheath rot, and yellow stem borer	India	[88]
	Hua 2B	Superior quality, stable traits	China	[113]
	AC-1	Saline-alkaline tolerance	Bangladesh	[114]
	Chuanhui 907	Superior quality, strong combining ability, good restoration ability, rice blast resistance	China	[115]
	CR Dhan 801 (CRAC2224–1041, IET18720), Phalguni	Resistance to leaf blast and gall midge, moderate resistance to leaf sheath rot, rice stripe virus, yellow stem borer, and brown spot	India	[89]
	Chuanhui 1618	Large panicle, superior quality, strong combining ability, good restoration ability, rice blast resistance	China	[116]
Hybrid rice	Miai 64S	Stable fertility, high yield, wide compatibility	China	[97]
	1103S, 8906S, 8902S	Stable infertility, practical value	China	[117]
	Liangyou 1178	High yield, superior quality, multi-resistance	China	[117]
	HS-1, HS-2, HS-3	Good economic traits, outcrossing characteristics and combining ability	China	[118]
	Hua 1A	Good outcrossing characteristics, high combining ability	China	[112]
	1286S, 6442S	Stable yield, high yield	China	[119]
	Jinshan S-1	Stable infertility, long infertility period, superior quality	China	[120]
	Huaxiang 7	High quality rice, high yield, moderate resistance to rice blast disease	China	[121]
	Xiang 125S	High quality rice with strong compatibility	China	[122]
	Hua 2A	Stable infertility, high outcrossing rate, superior quality	China	[113]
	V25S	High outcrossing seed-setting rate, superior quality	China	[123]
	EH1S	High outcrossing seed-setting rate, rice blast resistance, superior quality	China	[124]

## 5. Challenges in Rice Anther Culture Breeding

The anther culture breeding of rice not only shortens the breeding cycle and improves selection efficiency but also integrates with transgenic technique and molecular marker-assisted selection to develop new varieties with multiple resistances, widespread adaptability, high yield, and superior quality. However, the practical application of anther culture in rice breeding programs faces several challenges, and its full potential is constrained by various factors.

Genotype—the primary challenge in anther culture breeding—largely determines the efficiency of anther culture. Moreover, numerous steps, significant workload, long cycles, low callus induction, green plantlet regeneration rates, and lower indica rice anther culture efficiency are challenges encountered in the application of anther culture technique in rice breeding programs. To alleviate genotype constraints, previous studies have suggested selecting materials with high anther culture efficiency as parents or using indica–japonica hybrid progenies with a higher proportion of japonica pedigree to exploit heterosis and improve anther culture efficiency. Moreover, identifying major genes influencing anther culture efficiency and employing transgenic technique and molecular marker-assisted selection can alleviate genotype constraints.

Browning of the anthers and callus, as well as the occurrence of albino plantlets, also affect the efficiency of rice anther culture. Browning results from the activation of polyphenol oxidases in the cultured tissues, leading to other enzyme inactivation and growth inhibition [125]. Both overly young and old anthers are prone to browning, while high concentrations of inorganic salts and sugars in the culture medium can exacerbate callus browning. Albino plantlets, a recessive trait controlled by multiple loci [126], are associated with abnormal gene expression or mutations, and the absence of chloroplasts occurs under the combined action of environment and genes. Several measures can be taken to prevent the browning of the anthers and callus and the appearance of albino plantlets. To reduce browning, sampling young spikes whose anthers are at the mid–late uninucleate stage, optimizing culture conditions like temperature, light duration and light intensity, and adding anti-browning agents, such as activated carbon, PVP, vitamin C, and Na_2_S_2_O_3_, can be effective [127]. Zhang et al. [128] found that inserting the transposon into the BOC1 gene promoter lowered callus browning in rice. Studies have shown that reducing manganese content and inorganic salt concentrations in the callus induction medium and optimizing hormone ratios (e.g., KT or 2,4-D) [129] can lower albino plantlet rates and simultaneously enhance callus induction rates. However, chloroplasts are modulated by both nuclear and chloroplast genes, so researchers need to identify comprehensive solutions.

## 6. Prospects for Rice Anther Culture Breeding

Currently, genotype remains the most significant factor constraining the efficiency of anther culture in rice. As callus induction rate and green plantlet differentiation rate are quantitative traits, identifying major genes influencing anther culture efficiency is a critical method for overcoming genotype constraints. Molecular marker-assisted selection has already identified several quantitative trait loci (QTLs) associated with anther culture efficiency. With the maturation of gene editing and knockout technologies, future research can employ reverse genetics to edit or knockout genes with similar functions in other crops to construct mutant libraries, investigate the phenotypes of mutants, and validate them through forward genetics. This approach may expedite the discovery of major genes influencing anther culture efficiency, alleviate genotype constraints, and facilitate the utilization of anther culture technique for developing new germplasm resources.

In addition to in vitro anther culture, haploid plants can be generated through in vitro culture of microspores. The technique of using free pollen to perform microspore culture is well established in higher plants such as maize (*Zea mays* L.) [130], wheat (*Triticum aestivum* L.) [131], rapeseed (*Brassica campestris* L.) [132], and tobacco (*Nicotiana tabacum* L.) [133], but its application in rice remains underdeveloped. In vitro culture of microspores allows direct contact between pollen and the medium, accelerates the culture process, and reduces the consumption of experimental materials, offering theoretical advantages over traditional anther culture. Researchers can utilize the in vitro culture of microspores to breed new rice varieties in the future.

The development and utilization of new rice varieties with resistance to pests, diseases, and drought, as well as saline–alkali tolerance, high yield, and superior quality, can be facilitated by integrating anther culture with other technologies, such as transgenic technique and molecular marker-assisted selection. Standardizing and simplifying protocols for anther culture to minimize impacts from experimental operations, along with improving anther culture system in rice, will contribute to the development and utilization of new rice varieties and the enrichment of rice germplasm resources in China.
